# Influence of cervical treatment methods on subsequent vaginal lesions: A study of HPV-related neoplasia

**DOI:** 10.17305/bb.2024.10523

**Published:** 2024-12-01

**Authors:** Yuanyuan Chen, Haizhen He, Xiaoxi Wang, Yan Ye, Jiajia Pan

**Affiliations:** 1 Wenzhou People’s Hospital, The Third Clinical Institute Affiliated to Wenzhou Medical University, Wenzhou, China; 2 Department of Gynecological Protection, Wenzhou People’s Hospital, Wenzhou, China

**Keywords:** Cervical intraepithelial neoplasia, vaginal intraepithelial neoplasia (VaIN), human papillomavirus (HPV), menopausal status, treatment methods, risk analysis

## Abstract

The development of cervical and vaginal intraepithelial neoplasias (CIN and VaIN) is strongly associated with human papillomavirus (HPV) infections, representing key precancerous conditions in women. This study investigates the influence of different cervical treatment methods on the rate of subsequent vaginal neoplasia. It also considers age and menopausal status as risk factors for higher grade VaIN and the role of persistent HPV infections in the development of new VaIN cases post-treatment. The cohort consisted of 275 female patients treated for CIN, with a follow-up period of six months including HPV and ThinPrep cytologic test (TCT) testing. The evaluated treatments included laser therapy, cervical conization, loop electrosurgical excision procedure (LEEP), and radical hysterectomy. Statistical analysis was performed using SPSS 26.0 to determine treatment efficacy, the impact of age and menopausal status, and the relationship between HPV clearance and VaIN outcomes. Radical hysterectomy was linked with a higher recurrence of VaIN. Additionally, patients over 50 years old and those who were postmenopausal were significantly more likely to develop more severe VaIN and persistent HPV infections. The persistence of HPV after treatment was linked to a higher incidence of new VaIN cases. High-risk HPV (hrHPV) significantly increased the recurrence of VaIN, with no significant link found between TCT results and VaIN severity. Therefore, selecting appropriate cervical lesion treatment, considering the patient’s age and menopausal status, and managing HPV infections are essential in preventing and managing the risk and progression of VaIN. Radical hysterectomy showed a distinct increase in VaIN incidence, emphasizing the need for individualized clinical assessments.

## Introduction

Vaginal intraepithelial neoplasia (VaIN) represents a rare precancerous condition occurring within the female lower genital tract [1]. Increased attention to vaginal epithelial anomalies, coupled with advancements in detection methodologies, including cytology and human papillomavirus (HPV) testing, has led to a rise in the detection rate of VaIN in recent years [2–6]. VaIN is categorized into various grades, with VaIN 2/3 considered equivalent to high-grade cervical intraepithelial neoplasia (CIN 2/3) precancerous lesions, while VaIN1 (low-grade VaIN [7]) represents a benign manifestation of HPV infection [4, 8–11]. Beyond HPV infection, other known risk factors for VaIN and vaginal cancer include immunosuppression, smoking, multiple sexual partners, and early onset of sexual activity [10–14]. The prevalence of these risk factors, alongside the ubiquity of HPV infection, underscores the importance of research and treatment efforts targeting VaIN [15, 16].

Current treatment options for CIN include laser therapy, loop electrosurgical excision procedure (LEEP), cervical conization, and hysterectomy [14, 17, 18]. It has been reported that approximately 1%–7% of patients treated with hysterectomy for CIN may develop VaIN months to years post-surgery [19]. Notably, hysterectomy as a treatment for CIN has been identified as a risk factor for the development of VaIN [20]. However, the impact of different cervical lesion treatment methods on the incidence of post-CIN treatment VaIN remains unexplored mainly [21, 22]. Furthermore, studies have highlighted significant individual variations in the progression rate of vaginal lesions, underscoring the need for proactive treatment of patients with identified risk factors [19].

**Figure 1.**
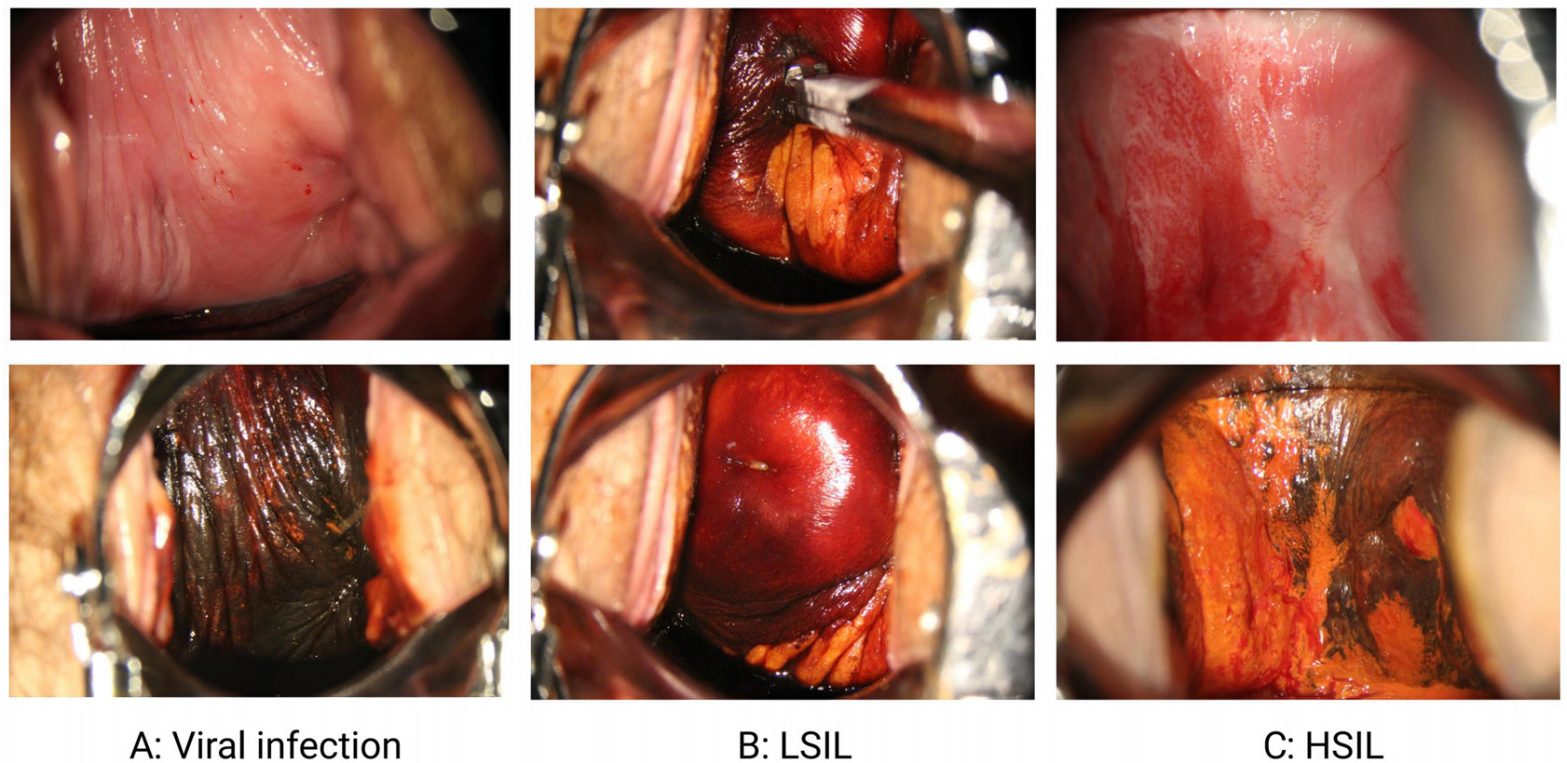
**Incidence of vaginal lesions post-cervical lesion treatment.** This figure illustrates the incidence of VaIN following CIN treatment, including the distribution of VaIN1+, as well as cases of patients with vaginal viral infection (A), LSIL (B), and HSIL (C). VaIN: Vaginal intraepithelial neoplasia; CIN: Cervical intraepithelial neoplasia; LSIL: Low-grade squamous intraepithelial lesion; HSIL: High-grade squamous intraepithelial lesion.

Despite extensive discussions on the treatment of CIN and VaIN, the specific mechanisms underlying the development of vaginal lesions post-cervical treatment, especially the pathological changes associated with VaIN in the context of high-risk HPV (hrHPV) infection, are still poorly understood [4, 23, 24]. As research deepens into the relationship between HPV infections, CIN, and subsequent VaIN recurrence, it becomes increasingly critical to determine the impact of specific HPV infection types on the risk of VaIN recurrence [25]. Recent studies indicate that 73.2% of VaIN2/3 cases involve a single HPV genotype, whereas 26.8% display multiple infections (20.8% are dual infections, 4.8% are triple infections, and 1.2% are quadruple infections) [26]. Moreover, while menopausal status and age are known to influence the progression of these lesions, the specific effects of these factors on the occurrence and progression of VaIN and their roles in clinical treatment and prevention strategies require further clarification [27]. Notably, the relationship between HPV infection status and the risk of developing VaIN post-cervical lesion treatment, including whether deviations in medical examination and macroscopic viral infection statuses warrant active intervention, remains a pressing research question [24, 28, 29].

This study aims to thoroughly examine the impact of various methods of treating cervical lesions on the subsequent incidence of VaIN. It also seeks to investigate the potential associations between treatment methods and factors, such as preoperative CIN grade, HPV infection subtype, HPV negativity six months after surgery, ThinPrep cytologic test (TCT) results, and menopausal status, with regard to VaIN recurrence following CIN treatment. Given that VaIN is typically widespread and subject to diagnostic discrepancies in medical examinations, clarifying these relationships is crucial for developing effective clinical interventions and treatment strategies to reduce the risk of VaIN recurrence in women after CIN treatment. Through this research, we aim to provide more precise treatment guidance for clinicians, particularly for patients with risk factors, hoping to effectively reduce the recurrence rate of VaIN and enhance patients’ quality of life and treatment outcomes.

## Materials and methods

### Study population and grouping

This study selected 275 female patients treated at the Wenzhou People’s Hospital in Zhejiang Province as subjects from June 2017 to June 2022. The age distribution of patients ranged from 22 to 80 years. They were divided into two groups: the first group consisted of 218 patients who developed VaIN after treatment for CIN, including 176 cases of vaginal low-grade squamous intraepithelial lesions (LSIL) (64%), 42 cases of vaginal high-grade squamous intraepithelial lesions (HSIL) (15.27%), and 32 cases of VaIN1+ (28.83%; defined as recurrence of VaIN [20]). The second group comprised 57 patients with vaginal virus infection, characterized by the presence of koilocytotic cells, of which 33 cases (57.89%) were hrHPV infections (Figure 1). Regarding cervical lesion treatment methods, the incidence of VaIN1+ post-treatment was recorded as follows: 27 cases (9.41%) after laser therapy, 24 cases (22.64%) after cervical conization, 80 cases (9.66%) after LEEP, and 55 cases (44.35%) after radical hysterectomy.

### Inclusion and exclusion criteria

Inclusion criteria include: 1) women aged 22–80; 2) complete medical records and follow-up data; 3) treatment for cervical lesions, including laser therapy, cervical conization, LEEP, or radical hysterectomy; and 4) diagnosis of VaIN or vaginal virus infection confirmed by colposcopy-directed biopsy.

Exclusion criteria include: 1) patients with significant comorbidities or poor physical condition precluding study participation; 2) patients with severe systemic or psychiatric illnesses that hinder treatment cooperation and follow-up; 3) pregnant or breastfeeding women; and 4) patients allergic to drugs or devices used in the study.

### Treatment modalities and follow-up

Treatment methods for cervical lesions encompassed laser therapy, cervical conization, LEEP, and radical hysterectomy. All participants were subjected to retesting for HPV using the Roche Cobas HPV Test and TCT (CytoRich red system, Hologic Inc., Marlborough, MA, USA) six months post-treatment.

### Ethical statement

Consent was obtained from each participant or their legal representative after ensuring a comprehensive understanding of the study content and procedures, and written informed consent was signed voluntarily. The study protocol was reviewed and approved by the Clinical Ethics Committee of Wenzhou People’s Hospital (No. KY-2021-301), ensuring compliance with the Declaration of Helsinki.

### Statistical analysis

Data analysis was conducted using SPSS software version 26.0 (IBM Corp., Armonk, NY, USA). Univariate analyses assessed the associations between cervical lesion grade, age, HPV infection, previous treatments, menopausal status, and HPV status six months post-surgery. Chi-square tests and Students’ *t*-tests were utilized for univariate analyses, and both univariate and multivariate logistic regression analyses were applied to evaluate the incidence of VaIN post-treatment for cervical lesions. Cumulative incidence analysis was used to estimate the incidence of VaIN following different treatment methods. A *P*-value of less than 0.05 was considered statistically significant. Graphs and charts were generated using GraphPad Prism software (GraphPad Software, San Diego, CA, USA).

## Results

### Impact of cervical lesion treatment methods on the incidence of VaIN

**Figure 2.**
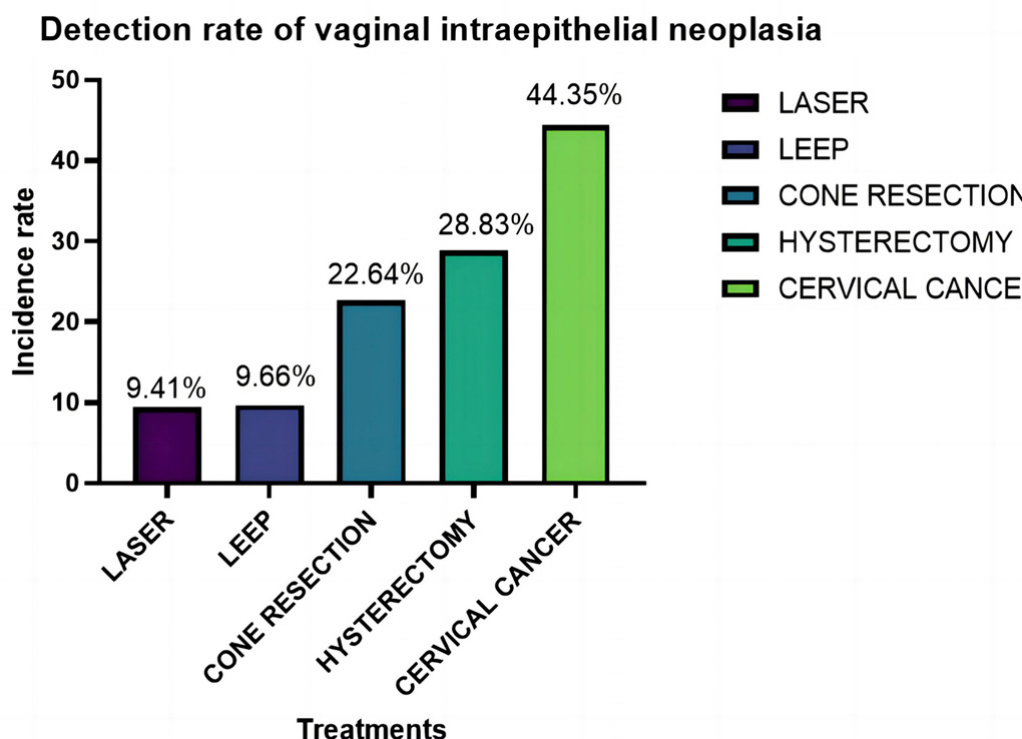
**Comparison of VaIN detection rates after treatment of cervical lesions, highlighting the effectiveness of different therapeutic approaches.** VaIN: Vaginal intraepithelial neoplasia; LEEP: Loop electrosurgical excision procedure.

This study investigates the influence of various cervical lesion treatment methods on the subsequent incidence of VaIN by comparing the rates of VaIN1+ occurrence following hysterectomy, laser therapy, LEEP, cervical conization, and radical hysterectomy. A total of 1456 female patients were included, with 111 undergoing hysterectomy, 287 receiving laser therapy, 828 treated with LEEP, 106 undergoing cervical conization, and 124 receiving radical hysterectomy for CIN. The findings revealed that 32 of the 111 CIN patients treated with hysterectomy (28.83%) developed VaIN1+; among the 287 patients treated with laser therapy, 27 (9.41%) developed VaIN1+; of the 828 patients treated with LEEP, 80 (9.66%) developed VaIN1+; and among the 124 patients treated with radical hysterectomy, 55 (44.35%) developed VaIN1+. Statistical analysis demonstrated significant differences in the incidence of VaIN following different treatment methods (*P <* 0.05) (Figure 2).

### Age distribution and lesion grade analysis in VaIN patients

**Figure 3.**
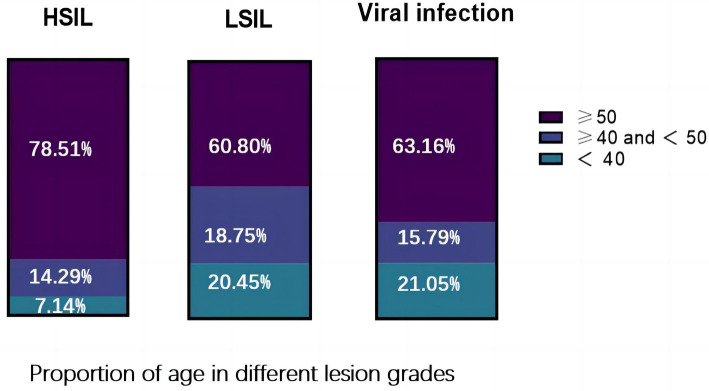
**Age distribution of patients with different categories of VaIN, providing insight into the prevalence and risk factors across various age groups.** VaIN: Vaginal intraepithelial neoplasia; LSIL: Low-grade squamous intraepithelial lesion; HSIL: High-grade squamous intraepithelial lesion.

The age distribution characteristics of VaIN and its relationship with lesion grades were assessed in 275 VaIN patients. The study examined the age distribution and proportions of patients with LSIL (VaIN1), HSIL, and those with viral infections to reveal the impact of age on the occurrence and progression of VaIN. Among the 275 VaIN patients included in this study, 176 (64%) were diagnosed with LSIL (VaIN1), 42 (15.27%) with HSIL, and 57 (20.73%) presented with viral infections. Specifically, the average age was 47.39 ± 11.29 years for LSIL patients, 53.83 ± 10.71 years for HSIL patients, and 46.42 ± 11.94 years for those with viral infections. Notably, in women over 50, the incidence of HSIL reached 78.51%, compared to 60.80% for LSIL and 63.16% for viral infections. This data significantly indicates that age is an essential factor influencing the occurrence and grade of VaIN lesions, with women over 50 being more susceptible to developing HSIL (*P <* 0.05) (Figure 3, Table 1). In summary, the results of this study highlight the significant role of age in the occurrence and progression of VaIN, especially indicating that women over 50 are more likely to develop higher grades of VaIN.

**Table 1:** Age distribution of patients with different types of VaIN

**Characteristics**	**Viral infection (*n*, %)**	**LSIL (*n*, %)**	**≥HSIL (*n*, %)**	**Total**
Age, mean (SD)	46.42 ± 11.94	47.39 ± 11.29	53.83 ± 10.71	
<40	12 (21.05)	37 (21.02)	3 (7.14)	52
≥40 and <50	9 (15.79)	53 (30.11)	6 (14.29)	68
≥50	36 (63.16)	86 (48.86)	33 (78.57)	155
***P* value**	*P >* 0.05	*P >* 0.05	*P >* 0.05	

LSIL: Low-grade squamous intraepithelial lesion; ≥HSIL: High-grade squamous intraepithelial lesion and more severe lesions; SD: Standard deviation; VaIN: Vaginal intraepithelial neoplasia.

### Incidence of VaIN and viral infections in postmenopausal women

**Figure 4.**
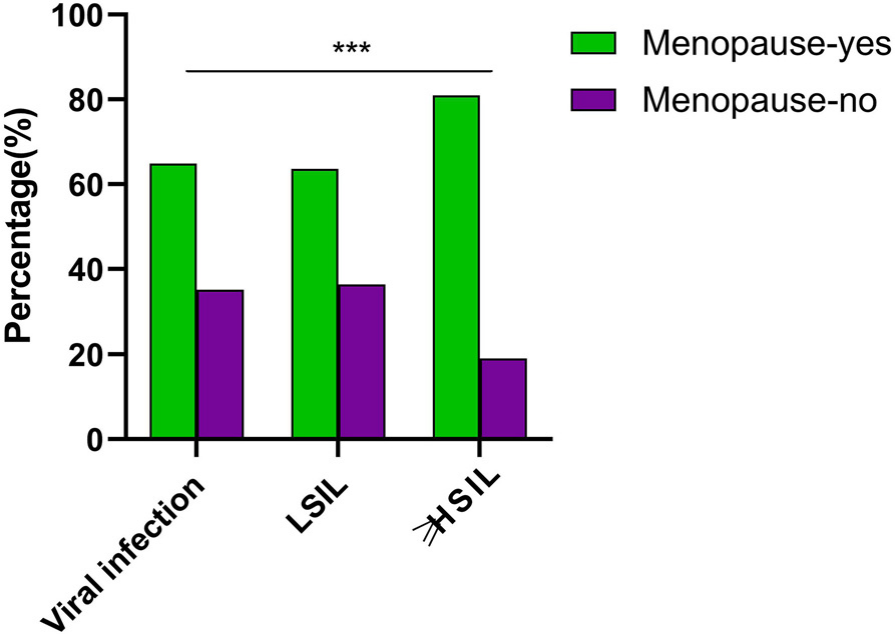
**Comparative analysis of the incidence of VaIN and viral infections in postmenopausal women.** This figure compares the incidence rates of VaIN and viral infections between postmenopausal and premenopausal women; ***P < 0.001. VaIN: Vaginal intraepithelial neoplasia; LSIL: Low-grade squamous intraepithelial lesion; ≥HSIL: High-grade squamous intraepithelial lesion and more severe lesions.

Given the physiological changes postmenopausal women undergo that may influence susceptibility to various diseases, including VaIN and viral infections [30], this section aims to assess the impact of menopausal status on the likelihood of VaIN and viral infections. The menopausal status of patients with epithelial lesions and viral infections was evaluated to determine the incidence rates of VaIN and viral infections within this demographic. Out of 275 female patients analyzed, 218 presented with VaIN, and 57 had viral infections. The analysis revealed that 146 (66.97%) of the VaIN patients were postmenopausal; among the 57 patients with viral infections, 37 (64.91%) were in a postmenopausal state. Statistical analysis of these data indicated a significant increase in the probability of VaIN and viral infections among postmenopausal women compared to their premenopausal counterparts (Table 2, Figure 4). In summary, the findings demonstrate elevated epithelial and viral infections in postmenopausal women, particularly noting a significant rise in high-grade lesions (≥HSIL). This finding underscores the importance of intensified detection and preventive measures for VaIN and viral infections among the postmenopausal female population.

**Table 2:** Association between menopausal status and the incidence of VaIN and viral infections

**Characteristics**	**Viral infection (*n*, %)**	**LSIL (*n*, %)**	**≥HSIL (*n*, %)**	**Total**	***P* value**
Menopause					*P <* 0.001
Yes	37 (64.91)	112 (63.64)	34 (80.95)	183	
No	20 (35.09)	64 (36.36)	8 (19.05)	92	

LSIL: Low-grade squamous intraepithelial lesion; ≥HSIL: High-grade squamous intraepithelial lesion and more severe lesions; VaIN: Vaginal intraepithelial neoplasia.

**Figure 5.**
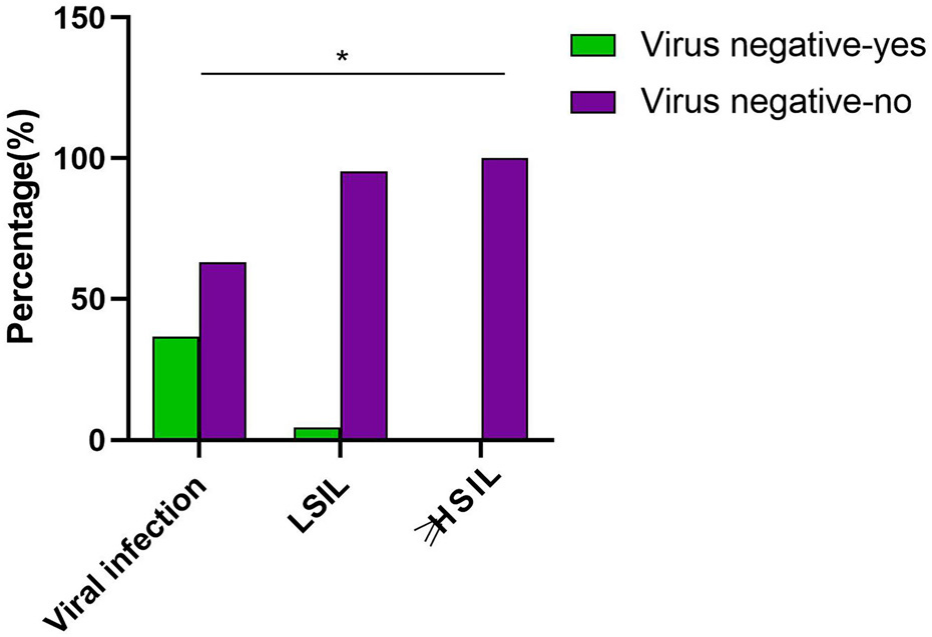
**Analysis of the relationship between HPV clearance status post-cervical lesion treatment and the risk of new VaIN occurrences.** This figure illustrates the association between HPV clearance status post-treatment of cervical lesions and the risk of new VaIN occurrences; *P < 0.05. VaIN: Vaginal intraepithelial neoplasia; HPV: Human papillomavirus; LSIL: Low-grade squamous intraepithelial lesion; ≥HSIL: High-grade squamous intraepithelial lesion and more severe lesions.

### Relationship between HPV clearance post-cervical lesion treatment and new VaIN occurrences

HPV infection is a leading cause of CIN and VaIN [31]. The change in HPV status post-treatment plays a critical role in preventing new occurrences of VaIN. This study investigates the association between HPV clearance following cervical lesion treatment and the incidence of new VaIN occurrences. It includes patients treated for cervical lesions, analyzing the relationship between HPV infection status six months post-treatment and new VaIN occurrences. The findings indicate that none of the patients diagnosed with vaginal HSIL showed HPV clearance after six months.

In contrast, among the LSIL patient group, 108 (95.45%) maintained their HPV-positive status post-treatment, while only 8 (4.55%) achieved HPV clearance. These results significantly demonstrate that the risk of new VaIN occurrences is closely linked to a persistent positive HPV status post-treatment, bearing statistical significance (*P <* 0.05) (Table 3, Figure 5). In summary, this study confirms that patients who do not achieve HPV clearance after treatment for cervical lesions are more likely to develop new occurrences of VaIN, especially evident among HSIL patients.

**Table 3:** Analysis of the correlation between the post-treatment HPV negative status and the occurrence of new VaIN in patients with cervical lesions

**Characteristics**	**Viral infection (*n*, %)**	**LSIL (*n*, %)**	**≥HSIL (*n*, %)**	**Total**	***P* value**
The virus turned negative 6 months after treatment					*P <* 0.05
Yes	21 (36.84)	8 (4.55)	0 (0)	29	
No	36 (63.16)	168 (95.45)	42 (100)	246	

LSIL: Low-grade squamous intraepithelial lesion; ≥HSIL: High-grade squamous intraepithelial lesion and more severe lesions; VaIN: Vaginal intraepithelial neoplasia.

### Association between HPV infection type and VaIN recurrence post-cervical lesion treatment

This study analyzed the HPV infection types in patients experiencing VaIN recurrence post-CIN treatment, examining the relationship between single and multiple HPV infections, as well as hrHPV infections and VaIN recurrence (Table 4). Among the 218 patients analyzed for VaIN recurrence post-CIN treatment, 121 (55.50%) had a single HPV infection, 94 (43.12%) had multiple HPV infections, and 201 (92.20%) were infected with hrHPV types. Notably, all patients with HSIL recurrence (≥HSIL) were infected with hrHPV types. Statistical analysis indicated no significant association between VaIN recurrence and multiple HPV infections (*P* > 0.05) but a significant correlation with hrHPV infections (*P <* 0.05), especially as 100% of HSIL patients harbored hrHPV infections. These findings highlight the critical role of hrHPV infections in VaIN recurrences, emphasizing that hrHPV significantly increases the risk of VaIN recurrence, particularly among HSIL patients.

**Table 4:** Clinicopathological features and HPV infection status in patients with recurrent VaIN after cervical lesion treatment

**Characteristics**	**Viral infection (*n*, %)**	**LSIL (*n*, %)**	**≥HSIL (*n*, %)**	**Total**	***P* value**
**HPV status**	46.42 ± 11. 94	47.39 ± 11. 29	53.83 ± 10. 71		*P <* 0.001
hrHPV-positive	12 (21.05)	37 (21.02)	3 (7.14)	52	
lrHPV-positive	9 (15.79)	53 (30.11)	6 (14.29)	68	
HPV-negative	36 (63.16)	86 (48.86)	33 (78.57)	155	
**HPV infection**					*P >* 0.05
Multiple HPV infection	15 (26.32)	77 (43.75)	17 (40.48)	109	
Single HPV infection	29 (50.86)	96 (54.55)	25 (59.52)	150	
Negative	13 (22.81)	3 (1.70)	0 (0)	16	
Total	57	176	42	275	

LSIL: Low-grade squamous intraepithelial lesion; ≥HSIL: High-grade squamous intraepithelial lesion and more severe lesions; HPV: Human papillomavirus; hrHPV: High-risk human papillomavirus; lrHPV: Low-risk human papillomavirus; VaIN: Vaginal intraepithelial neoplasia.

**Figure 6.**
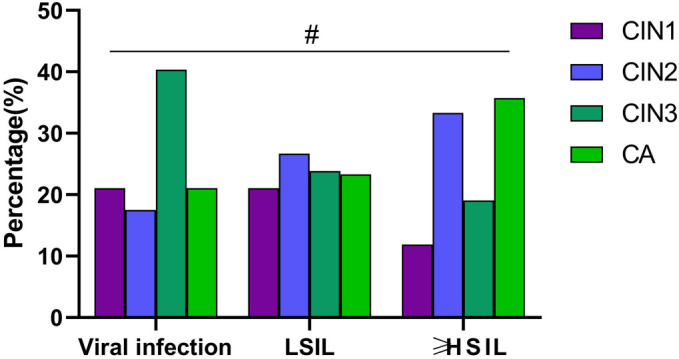
**Analysis of the association between cervical lesion grade and the risk of recurrence of new VaIN occurrences.** This figure compares the risk of new VaIN occurrences recurrence post-treatment among patients with different grades of cervical lesions, including analyses for CIN1, CIN2, CIN3, and CA patients; ^#^*P* > 0.05. VaIN: Vaginal intraepithelial neoplasia; CIN: Cervical intraepithelial neoplasia; LSIL: Low-grade squamous intraepithelial lesion; ≥HSIL: High-grade squamous intraepithelial lesion and more severe lesions; CA: Cervical cancer.

### Association analysis between cervical lesion history and recurrence of new VaIN occurrences

The recurrence of VaIN may be influenced by several factors, including the grade of prior cervical lesions, which could be a crucial consideration [32]. This study explored the relationship between new VaIN occurrences post-cervical lesion treatment and the grade of previous cervical lesions to assess any clear correlation. Patients who developed new VaIN occurrences post-treatment were analyzed based on the grade of their prior cervical lesions (Table 5). Specifically, 51 patients (23.39%) had prior CIN1, 61 (27.98%) had CIN2, 50 (22.94%) had CIN3, and 56 (25.69%) were cervical cancer (CA) patients. Statistical analysis attempted to reveal the association between VaIN recurrence and the grade of cervical lesions before treatment. The analysis showed that while VaIN recurrence is related to the patient’s history of cervical lesions, the incidence rate does not significantly correlate with the specific grade of prior CIN (*P* > 0.05) (Figure 6). This finding suggests that in clinical practice, irrespective of the previous cervical lesion grade, equal monitoring and prevention strategies should be applied to all post-treatment patients to minimize the risk of VaIN recurrence.

**Table 5:** Clinical characteristic analysis of the relationship between cervical lesion grade and new onset VaIN recurrence

**Characteristics**	**Viral infection (*n*, %)**	**LSIL (*n*, %)**	**≥HSIL (*n*, %)**	**Total**	***P* value**
Previous grade of cervical lesion					*P >* 0.05
CIN1	12 (21.05)	46 (21.05)	5 (11.90)	63	
CIN2	10 (17.54)	47 (26.70)	14 (33.33)	71	
CIN3	23 (40.35)	42 (23.86)	8 (19.05)	73	
CA	12 (21.05)	41 (23.30)	15 (35.71)	68	

LSIL: Low-grade squamous intraepithelial lesion; ≥HSIL: High-grade squamous intraepithelial lesion and more severe lesions; CIN: Cervical intraepithelial neoplasia; CA: Cervical cancer; VaIN: Vaginal intraepithelial neoplasia.

**Figure 7.**
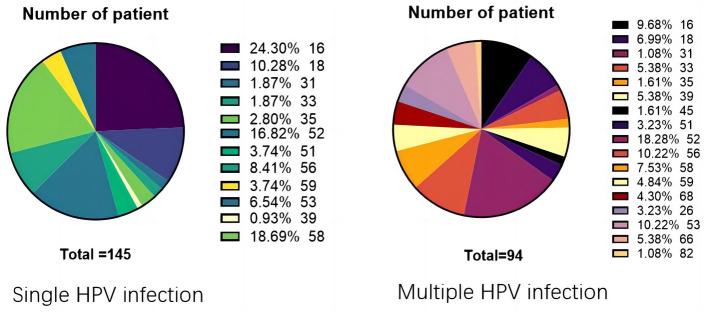
**Distribution of HPV types in female vaginal epithelial lesions.** HPV: Human papillomavirus.

### Analysis of the association between HPV types and vaginal epithelial lesions

Given the well-established link between HPV infection and the development of both cervical and vaginal epithelial lesions [33], this study aims to delve into the distribution and epidemiological characteristics of different HPV types within VaIN. By conducting a detailed analysis of patients with both single and multiple HPV infections, this research seeks to uncover the relationship between specific HPV types and vaginal epithelial lesions. Among the 239 female patients included, 145 exhibited single HPV infections, while 94 had multiple HPV infections. A detailed examination revealed that infection rates for HPV types 16, 18, 52, 53, 56, and 58 were significantly higher in single HPV infection cases than other types, indicating a strong association between these specific HPV types and vaginal epithelial lesions. Conversely, HPV52 was identified as the most prevalent type among patients with multiple HPV infections. These findings, presented in Figure 7, detail the distribution of different HPV types in vaginal epithelial lesions, highlighting the predominance of HPV16, 18, 52, 53, 56, and 58 in single HPV infections and the high prevalence of HPV52 in multiple HPV infections. The results underscore a significant association between specific HPV types and vaginal epithelial lesions.

### TCT results and their association with VaIN grades

The TCT has been widely adopted as a detection tool for CA and its precursor lesions [34]. However, its effectiveness in identifying VaIN remains uncertain. This study aims to explore the relationship between TCT results and different VaIN grades to assess the practical value of TCT in VaIN detection. TCT results indicated that 111 women (50.92%) were found to be in a state of no intraepithelial lesion or malignancy (NILM). Furthermore, 60 women (27.52%) were classified under atypical squamous cells of undetermined significance (ASC-US), while four women (1.83%) were in a condition where high-grade lesions, such as atypical squamous cells, cannot exclude HSIL (ASC-H). Additionally, 32 women (14.68%) were diagnosed with LSIL, and 11 women (5.05%) had HSIL. In total, 275 patients were analyzed to examine the correlation between TCT results and VaIN grades (Table 6). Patients were categorized into NILM, ASC-US, LSIL, HSIL, and ASC-H groups based on their TCT outcomes. The analysis revealed no significant difference in TCT detection rates across different VaIN grades, specifically NILM in 31 cases (54.39%), ASC-US in 17 cases (29.82%), LSIL in 6 cases (10.53%), HSIL in 3 cases (5.26%), and ASC-H in 0 cases. These results indicate that TCT outcomes are unreliable for detecting VaIN (*P* > 0.05). Therefore, the study concludes that TCT detection rates do not significantly correlate with lesion grades, emphasizing that in cases of hrHPV infections, even if TCT results indicate NILM, targeted diagnostic methods such as colposcopy should be employed to rule out VaIN and enhance diagnostic accuracy.

**Table 6:** Comparison of TCT results in patients with different grades of VaIN

**TCT**	***N***	**Pathology, *n* (%)**			***P* value**
		**Viral infection**	**LSIL**	**≥HSIL**	
NILM	142	31 (54.39)	88 (50)	23 (54.76)	*P >* 0.05
ASC-US	77	17 (29.82)	52 (29.55)	8 (19.05)	
LSIL	38	6 (10.53)	26 (14.77)	6 (14.29)	
HSIL	14	3 (5.26)	6 (3.41)	5 (11.90)	
ASC-H	4	0	4 (2.27)	0	
Total	275	57	176	42	

TCT: ThinPrep cytologic test; ASC-US: Atypical squamous cells of undetermined significance; LSIL: Low-grade squamous intraepithelial lesion; ≥HSIL: High-grade squamous intraepithelial lesion and more severe lesions; VaIN: Vaginal intraepithelial neoplasia; NILM: No intraepithelial lesion or malignancy.

**Figure 8.**
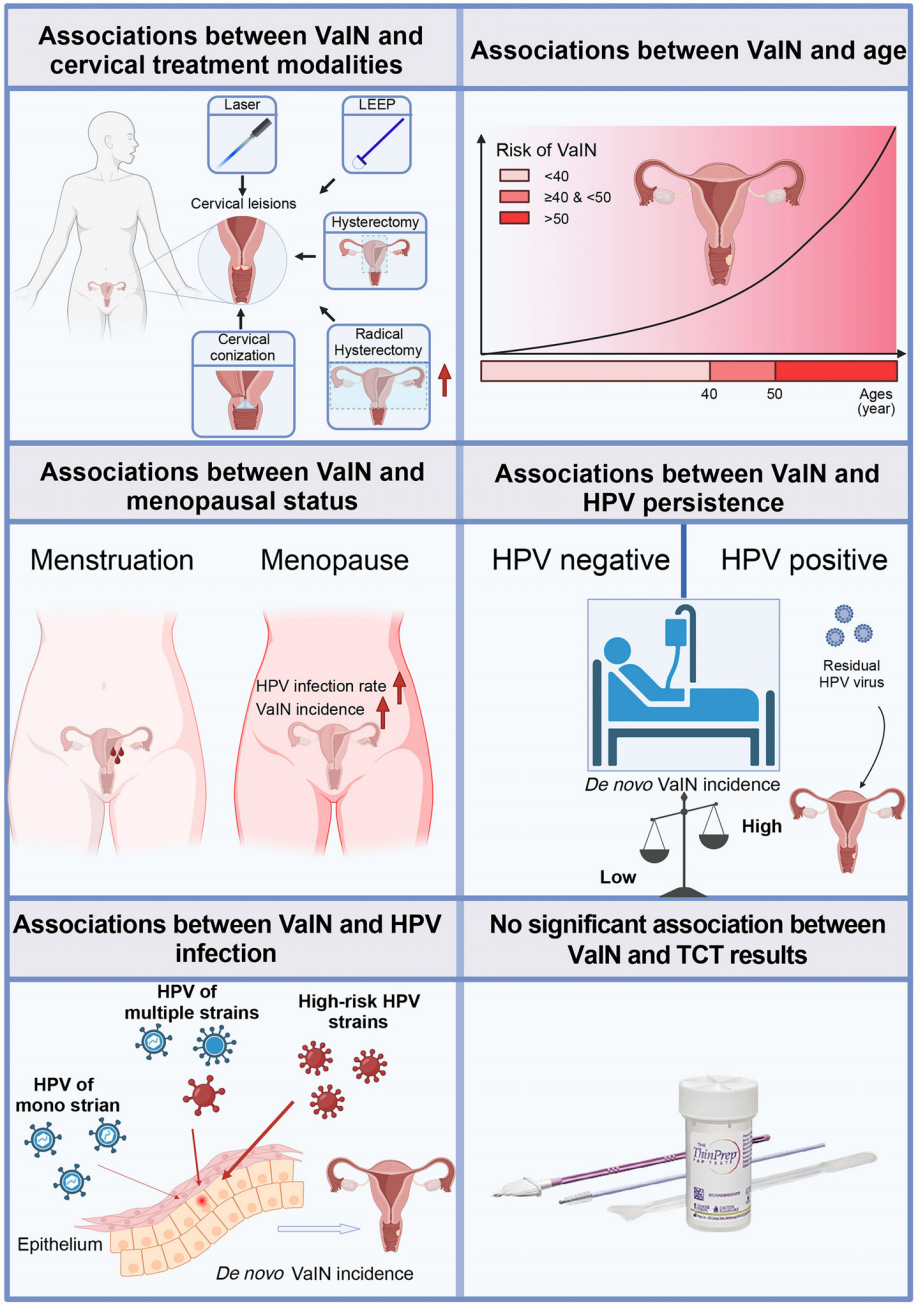
**Correlations in VaIN: treatment approaches, age, menopausal status, and HPV infections.** VaIN: Vaginal intraepithelial neoplasia; HPV: Human papillomavirus; TCT: ThinPrep cytologic test.

## Discussion

Despite advances in the treatment of cervical precancerous conditions, the mechanisms underlying the transition from CIN to VaIN in the context of HPV infections are not fully understood. This study provides a comprehensive analysis of the incidence of VaIN following treatment for CIN and its association with HPV infection. Our findings extend existing research by categorizing VaIN, a precancerous lesion, into grades I–III based on the degree of atypical proliferation observed in squamous cells. The study identifies several risk factors influencing the progression and persistence of these lesions, including infection with hrHPV, prior instances of CIN and CA, and the impact of surgical interventions such as hysterectomy [16, 35, 36]. These findings contribute to a deeper understanding of the dynamics between cervical treatments and subsequent vaginal epithelial changes.

Of note, our research emphasizes the need for personalized treatment strategies for VaIN, particularly highlighting that VaIN1, often transiently caused by lrHPV or hrHPV infections, typically resolves within two years. Conversely, HSIL is caused by persistent, transforming hrHPV infections, posing a greater risk of recurrence and progression to invasive vaginal cancer [37]. This evidence underscores the importance of proactive VaIN1 post-cervical lesion treatment to prevent its progression. We also focused on the notably increased risk of VaIN and viral infections among postmenopausal women, consistent with previous studies [38, 39], underscoring the influence of menopausal status on susceptibility to VaIN and HPV infections. Surprisingly, our findings indicate a higher incidence of VaIN1+ following radical hysterectomy, possibly due to anatomical changes post-surgery or the surgery’s failure to completely eradicate HPV infections. This finding contrasts with previous studies, which suggest that LEEP or laser treatments carry a relatively lower risk of VaIN [40].

Furthermore, our study examined the relationship between HPV clearance status and the risk of new VaIN occurrences, discovering that patients who did not achieve HPV clearance post-cervical lesion treatment were more likely to develop new VaIN occurrences. This finding underscores the significance of persistent HPV infection in the development of VaIN and highlights the importance of regular HPV detection following cervical lesion treatment to assess the risk of VaIN development. Interestingly, our study found no significant correlation between TCT results and VaIN grades, contrary to previous studies that suggested TCT as an effective tool for predicting VaIN grades. This discrepancy could be attributed to differences in sample sizes, study designs, or TCT methodologies, suggesting that the utility of TCT in VaIN detection requires further investigation and validation.

This research provides new insights for managing VaIN following CIN treatment, particularly highlighting the importance of individualized assessment and management post-CIN treatment. It supports the strategy of regular HPV screenings to assess VaIN risk [41], offering critical guidance for clinicians. These findings contribute to improving VaIN screening, prevention, and treatment strategies, thereby reducing patient morbidity. Despite its valuable insights, this study has limitations due to its retrospective design, which might introduce information and selection biases, limiting the causal interpretation of the results. The relatively small sample size and the study’s confinement to a single regional hospital might affect the generalizability and extrapolation of the findings. Additionally, the study did not thoroughly analyze the relationship between different HPV subtypes and VaIN risk, potentially overlooking subtype-specific risks. Finally, the lack of a control group precluded direct comparison of different treatment strategies on VaIN risk. Future research should adopt prospective, multicentric designs with larger sample sizes to address these limitations.

In light of these limitations, future research should focus on several key areas. Prospective, multicentric studies are needed to verify our findings and explore the relationship between different HPV subtypes and VaIN risk, helping to more accurately identify high-risk patient groups for targeted prevention and treatment strategies. Research should also evaluate the long-term effects of different treatment methods (e.g., LEEP, laser treatment, and radical hysterectomy) on HPV clearance rates and VaIN risk to guide clinical treatment decisions. Additionally, exploring potential interventions such as postmenopausal hormone replacement therapy in reducing VaIN risk might offer new preventative strategies for high-risk patients. Finally, future studies should explore emerging technologies (e.g., genomics and proteomics) to identify early biomarkers of VaIN, potentially aiding in early diagnosis and personalized treatment. Through these efforts, significant advancements in the prevention, diagnosis, and treatment of VaIN can be expected, thereby improving patient clinical outcomes.

## Conclusion

**Figure 9.**
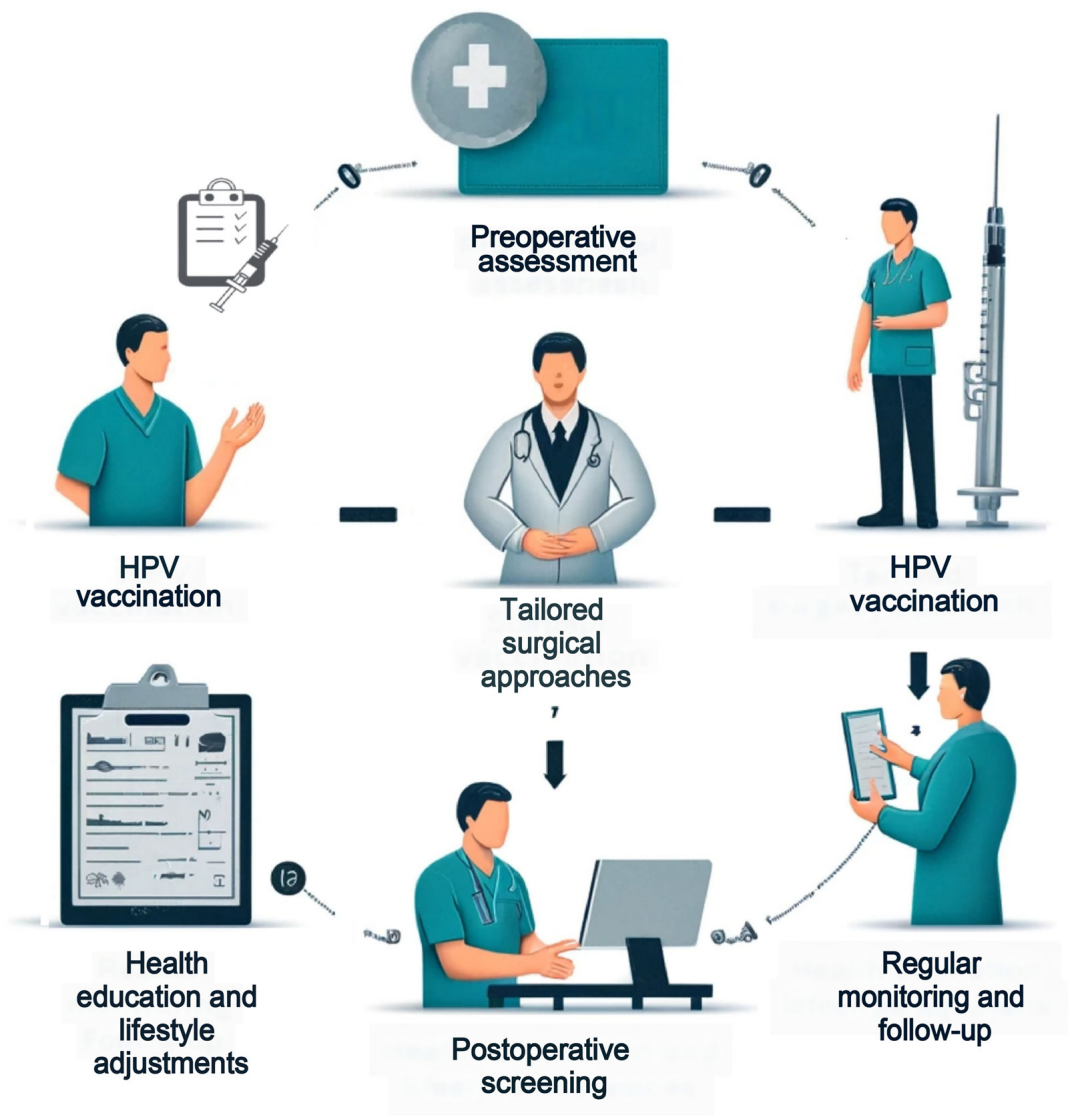
**A preliminary roadmap on preventing VaIN after a hysterectomy.** VaIN: Vaginal intraepithelial neoplasia; HPV: Human papillomavirus.

This study conducts a retrospective analysis of female patients treated with various cervical lesion treatment methods to explore the incidence of VaIN post-treatment and its association with HPV infection (Figure 8). The study unveiled several pivotal findings: notably, there exists a significant differential in the rates of VaIN following different treatments for cervical lesions, with radical hysterectomy exhibiting a substantially higher incidence of VaIN1+ compared to other modalities such as laser therapy and LEEP. Moreover, the onset of VaIN is closely linked with patient age, with individuals over the age of 50 being particularly susceptible to high-grade VaIN. Additionally, postmenopausal women demonstrate a marked increase in both VaIN and viral infections. A strong correlation was also established between hrHPV infections and the recurrence rate of VaIN, especially since all cases of recurrent high-grade VaIN were associated with hrHPV strains. These insights underscore the critical importance of screening for HPV post-treatment of cervical lesions and bolstering monitoring and preventative measures against VaIN in individuals with hrHPV infections. Such measures include regular HPV vaccinations and increased frequency of hrHPV strain screenings, particularly for patients who have undergone radical hysterectomy (Figure 9).

## Data Availability

All data can be provided as needed.

## References

[1] Borella F, Gallio N, Mangherini L (2023). Recent advances in treating female genital human papillomavirus related neoplasms with topical imiquimod. J Med Virol.

[2] Cong Q, Song Y, Wang Q, Zhang H, Gao S, Sui L (2018). A Retrospective Study of Cytology, High-Risk HPV, and Colposcopy Results of Vaginal Intraepithelial Neoplasia Patients. Biomed Res Int.

[3] Hodeib M, Cohen JG, Mehta S (2016). Recurrence and risk of progression to lower genital tract malignancy in women with high grade VAIN. Gynecol Oncol.

[4] Perkins RB, Wentzensen N, Guido RS, Schiffman M (2023). Cervical Cancer Screening: a Review. JAMA.

[5] Jhingran A (2022). Updates in the treatment of vaginal cancer. Int J Gynecol Cancer.

[6] Alrajjal A, Pansare V, Choudhury MSR, Khan MYA, Shidham VB (2021). Squamous intraepithelial lesions (SIL: LSIL, HSIL, ASCUS, ASC-H, LSIL-H) of Uterine Cervix and Bethesda System. Cytojournal.

[7] Monti E, Matozzo CMM, Cetera GE (2023). Correlation Between Colposcopic Patterns and Histological Grade of Vaginal Intraepithelial Neoplasia: a Retrospective Cohort Study [published correction appears in Anticancer Res 2023 Nov;43(11):5273]. Anticancer Res.

[8] Saslow D, Solomon D, Lawson HW (2012). American Cancer Society, American Society for Colposcopy and Cervical Pathology, and American Society for Clinical Pathology screening guidelines for the prevention and early detection of cervical cancer. Am J Clin Pathol.

[9] Darragh TM, Colgan TJ, Thomas Cox J (2013). The Lower Anogenital Squamous Terminology Standardization project for HPV-associated lesions: background and consensus recommendations from the College of American Pathologists and the American Society for Colposcopy and Cervical Pathology [published correction appears in Int J Gynecol Pathol 2013 Jul;32(4):432] [published correction appears in Int J Gynecol Pathol 2013 Mar;32(2):241]. Int J Gynecol Pathol.

[10] Kamolratanakul S, Pitisuttithum P (2021). Human Papillomavirus Vaccine Efficacy and Effectiveness against Cancer. Vaccines (Basel).

[11] Kechagias KS, Kalliala I, Bowden SJ (2022). Role of human papillomavirus (HPV) vaccination on HPV infection and recurrence of HPV related disease after local surgical treatment: systematic review and meta-analysis. BMJ.

[12] Daling JR, Madeleine MM, Schwartz SM (2002). A population-based study of squamous cell vaginal cancer: HPV and cofactors. Gynecol Oncol.

[13] Field A, Bhagat N, Clark S, Speed T, Razvi K (2020). Vaginal Intraepithelial Neoplasia: a Retrospective Study of Treatment and Outcomes Among a Cohort of UK Women. J Low Genit Tract Dis.

[14] Kesic V, Carcopino X, Preti M (2023). The European Society of Gynaecological Oncology (ESGO), the International Society for the Study of Vulvovaginal Disease (ISSVD), the European College for the Study of Vulval Disease (ECSVD), and the European Federation for Colposcopy (EFC) consensus statement on the management of vaginal intraepithelial neoplasia. Int J Gynecol Cancer.

[15] Ao M, Zheng D, Wang J, Gu X, Xi M (2021). Risk factors analysis of persistence, progression and recurrence in vaginal intraepithelial neoplasia [published correction appears in Gynecol Oncol 2022 Aug;166(2):369]. Gynecol Oncol.

[16] Yu D, Qu P, Liu M (2021). Clinical presentation, treatment, and outcomes associated with vaginal intraepithelial neoplasia: a retrospective study of 118 patients. J Obstet Gynaecol Res.

[17] Mosseri J, Hocquemiller R, Mergui JL, Uzan C, Canlorbe G (2022). Laser conization for cervical intraepithelial neoplasia: Effectiveness and obstetric outcomes. J Gynecol Obstet Hum Reprod.

[18] Athanasiou A, Veroniki AA, Efthimiou O (2022). Comparative effectiveness and risk of preterm birth of local treatments for cervical intraepithelial neoplasia and stage IA1 cervical cancer: a systematic review and network meta-analysis [published correction appears in Lancet Oncol 2022 Aug;23(8):e370]. Lancet Oncol.

[19] Iacobone AD, Radice D, Guerrieri ME (2023). Which Risk Factors and Colposcopic Patterns Are Predictive for High-Grade VAIN? A Retrospective Analysis. Diagnostics (Basel).

[20] Kim JH, Kim J, Kim K, No JH, Kim YB, Suh DH (2022). Risk Factor and Treatment of Vaginal Intraepithelial Neoplasia After Hysterectomy for Cervical Intraepithelial Neoplasia. J Low Genit Tract Dis.

[21] Yadav G, Srinivasan G, Jain A (2024). Cervical cancer: Novel treatment strategies offer renewed optimism. Pathol Res Pract.

[22] Liu Y, Huang N, Xu W (2022). A modified tracheal transection approach for cervical esophageal lesion treatment: a report of 13 cases. Front Surg.

[23] Cooper DB, Dunton CJ (2023). Colposcopy. In: StatPearls. Treasure Island (FL): StatPearls Publishing; November 12,.

[24] Fonseca BO, Possati-Resende JC, Salcedo MP (2021). Topical Imiquimod for the Treatment of High-Grade Squamous Intraepithelial Lesions of the Cervix: a Randomized Controlled Trial. Obstet Gynecol.

[25] Qiao YL, Wu T, Li RC (2020). Efficacy, Safety, and Immunogenicity of an Escherichia coli-Produced Bivalent Human Papillomavirus Vaccine: an Interim Analysis of a Randomized Clinical Trial. J Natl Cancer Inst.

[26] Preti M, Boldorini R, Gallio N (2024). Human papillomavirus genotyping in high-grade vaginal intraepithelial neoplasia: A multicentric Italian study. J Med Virol.

[27] Dang X, Lu Q, Li J (2024). Exploring the potential prompting role of cervical human papilloma virus detection in vulvar lesions: a cross-sectional study in China. Front Oncol.

[28] Pinkiewicz M, Dorobisz K, Zatoński T (2022). Human Papillomavirus-Associated Head and Neck Cancers. Where are We Now? A Systematic Review. Cancer Manag Res.

[29] Nguyen HDT, Le TM, Lee E (2023). Relationship between Human Papillomavirus Status and the Cervicovaginal Microbiome in Cervical Cancer. Microorganisms.

[30] Liu Y, Li Z (2024). Vaginal pH value can affect the susceptibility to human papillomavirus infection. BMC Infect Dis.

[31] Zhang S, Saito M, Okayama K (2021). HPV Genotyping by Molecular Mapping of Tissue Samples in Vaginal Squamous Intraepithelial Neoplasia (VaIN) and Vaginal Squamous Cell Carcinoma (VaSCC). Cancers (Basel).

[32] Buchanan TR, Zamorano AS, Massad LS (2019). Risk of cervical and vaginal dysplasia after surgery for vulvar intraepithelial neoplasia or cancer: a 6 year follow-up study. Gynecol Oncol.

[33] Lin CY, Wang CC, Wu RC (2021). Inhibition of BIRC2 Sensitizes α7-HPV-Related Cervical Squamous Cell Carcinoma to Chemotherapy. Int J Mol Sci.

[34] Ma JH, You SF, Xue JS (2022). Computer-aided diagnosis of cervical dysplasia using colposcopic images. Front Oncol.

[35] Inturrisi F, Rozendaal L, Veldhuijzen NJ, Heideman DAM, Meijer CJLM, Berkhof J (2022). Risk of cervical precancer among HPV-negative women in the Netherlands and its association with previous HPV and cytology results: a follow-up analysis of a randomized screening study. PLoS Med.

[36] Inturrisi F, Bogaards JA, Siebers AG, Meijer CJLM, Heideman DAM, Berkhof J (2022). Women with a positive high-risk human papillomavirus (HPV) test remain at increased risk of HPV infection and cervical precancer ≥15 years later. Tumour Virus Res.

[37] Li A, Li J, Austin RM (2021). Aptima HPV messenger RNA testing and histopathologic follow-up in women with HSIL cytology: a study emphasizing additional review of HPV-negative cases. Cancer Cytopathol.

[38] Sasagasako N, Kosaka K, Sagae Y (2020). Recurrent vaginal intraepithelial neoplasia successfully treated with topical imiquimod: a case report. Mol Clin Oncol.

[39] Zhang YY, Xia R, Chen D, Zhang X (2022). Analysis of related factors of cervical intraepithelial neoplasia complicated with vaginal intraepithelial neoplasia. Clin Transl Oncol.

[40] Parra-Herran C,  Malpica A, Oliva E, Zannoni GF, Ramirez PT, Rabban JT (2021). Endocervical Adenocarcinoma, Gross Examination, and Processing, Including Intraoperative Evaluation: Recommendations From the International Society of Gynecological Pathologists. Int J Gynecol Pathol.

[41] Mitra A, MacIntyre DA, Marchesi JR, Lee YS, Bennett PR, Kyrgiou M (2016). The vaginal microbiota, human papillomavirus infection and cervical intraepithelial neoplasia: what do we know and where are we going next?. Microbiome.

